# Evaluating the corporate social responsibility agenda for high-cost novel therapies: roles for government and civil society

**DOI:** 10.1186/s12961-025-01421-w

**Published:** 2025-11-28

**Authors:** Anna Wong, Gul Saeed, Sarah Garner, Jillian Kohler

**Affiliations:** 1 University of Toronto Leslie Dan Faculty of Pharmacy, Toronto, Canada; 2 Yale School of Public Health, New Haven, United States of America; 3 World Health Organization Regional Office for Europe, Copenhagen, Denmark; 4 Consultant, London, United Kingdom; 5 University of Toronto Dalla Lana School of Public Health, Toronto, Canada; 6 University of Toronto Munk School of Global Affairs and Public Policy, Toronto, Canada

**Keywords:** Access to medicines, Corporate social responsibility, Corporate determinants of health

## Abstract

**Background:**

Corporate social responsibility (CSR) activity in the pharmaceutical industry is frequently directed towards improving patient access to medicines amongst low-income populations. This research reports on findings from a mixed literature and key informant study of pharmaceutical sector CSR activity and its applicability in the high-cost novel therapeutics space.

**Methods:**

Academic and grey literature documents were extracted from online databases in a rapid literature review, focusing on four key areas of interest: (i) CSR or benefit company activity, (ii) the pharmaceutical industry, (iii) the development and sale of high-cost novel medicines and (iv) the role of government and civil society in this space. Ten semistructured interviews amongst key informants, including medical activists, pharmaceutical industry representatives, patient advocates, employees at nongovernmental organizations (NGOs), consultants for international organizations and academic researchers were also conducted related to these topics.

**Results:**

We find that CSR strategies vary depending on partner identity and country ability to pay. Differential pricing schemes and flexible patent approaches tend to be pursued unilaterally by companies, whereas companies frequently partner with local private sector, government, nongovernmental organizations and academic actors when implementing patient support programs, medicines donations, medicines delivery programs and rare and neglected disease research and development (R&D) initiatives. Patient support programs are more prevalent in high-income countries with minimal state-subsidized healthcare, whilst differential and tiered pricing strategies are more frequently pursued in lower-income countries.

**Conclusions:**

Pharmaceutical CSR strategies may benefit from greater coordination with government and civil society actors. Opportunities for government and civil society actors to take an active role in better aligning CSR activity with patient needs and universal health coverage include promoting greater adoption of alternative corporate structures and providing active external recognition of successful CSR initiatives through reputational and funding awards.

## Background

Corporate social responsibility (CSR) refers to a management practice whereby for-profit companies integrate social and/or environmental priorities into their business operations and stakeholder interactions [1]. The modern paradigm of CSR originated at the 1992 Earth Summit, where a voluntary private sector approach to environmental protection and sustainable development was endorsed as a complement to direct government regulation of industry actors [2]. Underlying the CSR movement is the economic proposition that for-profit firms incur political and social operating costs distinct from traditionally conceptualized business costs, and that these costs can be addressed by good corporate stewardship of the communities and environments in which they operate [3]. Whilst CSR is viewed differently around the world depending on cultural and historical circumstances, global interest in the phenomenon and its role in promoting sustainable development has led to the proliferation of international CSR guidelines including the United Nations (UN) Global Compact, the Global Reporting Initiative Guidelines, the UN Ruggie Principles and the European Union (EU) Corporate Governance Code.

The early rise of CSR coincides with a period in time in which multinational companies, including some pharmaceutical companies, faced increasing criticism by the public for failing to align their business interests with the best interests of society at large [4]. For example, cited roots of public distrust in the pharmaceutical industry have included the promotion of drugs that prioritize short-term health benefits over long-term cures, the concealment of significant and sometimes deadly adverse product effects, a neglect of certain patient groups when developing new therapeutic products, a general lack of transparency and the conscious pursuit of product prices that are unaffordable to many patients [5].

The pharmaceutical sector is amongst the most heavily regulated commercial sectors. It is subject to international and domestic laws and regulations governing aspects including product competition, distribution, marketing, quality, safety and efficacy. However, there is considerable variation in the implementation and enforcement of these laws and regulations, as well as many aspects of medicines access that are not directly subject to government regulation. Aligning with UN Sustainable Development Goal 3.8 (achieving universal health coverage, including access to safe, effective quality and affordable essential medicines and vaccines for all), CSR activity in the pharmaceutical industry is frequently directed towards improving patient access to medicines amongst populations unable to afford costly new innovative products.

In 2020, the WHO Regional Office for Europe, in collaboration with the Norwegian Ministry of Health and Care Services and the Norwegian Medicines Agency, launched the Oslo Medicines Initiative (OMI) to define clear avenues for public and civil society support of this activity [6]. On the basis of the principles of solidarity, transparency and sustainability, the work of the OMI culminated by creating the WHO Regional Office for Europe’s Access to Novel Medicines Platform (NMP), a formal stakeholder collaboration platform designed to improve affordable and equitable access to effective, novel, high-priced medicines within the WHO European Region [6].

The NMP has four main strategic aims: (1) to establish a formal collaboration mechanism to promote dialogue and knowledge exchange between Member States, non-state actors, partners and other stakeholders to improve access to effective high-cost novel medicines; (2) to implement actions which improve pharmaceutical market transparency, build trust and facilitate accountability; (3) to support voluntary collaborations to promote patient access focussed on solidarity, including horizon scanning, demand aggregation and lifecycle management; and (4) to develop methods, indicators and systems that enable risk sharing and good governance within the pharmaceuticals market [6].

This study, originally developed as a concept note for the WHO Regional Office for Europe Division of Country Health Policies and Systems, reports on findings from a rapid review of the literature and a targeted sample of key informant interviews investigating current pharmaceutical sector CSR activities directed towards improving access to high-cost novel therapies (i.e. newly approved non-generic prescription medicines with high launch prices), as well as the roles of different stakeholders in ensuring equitable and sustainable access to these medicines. It reviews ongoing areas of public and civil society collaboration with pharmaceutical sector actors and highlights promising areas for future engagement directed towards the NMP’s four strategic aims, particularly in terms of opportunities for government and civil society participation.

## Methods

English-language academic and grey literature documents were extracted from the online databases Medline, Embase, PubMed, Scopus, Web of Science and Google Scholar, as well as targeted global health, economic development and pharmaceutical company websites in January 2023. The literature search string focussed on four key areas of interest: (i) CSR or benefit company activity, (ii) the pharmaceutical industry, (iii) the development and sale of high-cost novel medicines and (iv) the role of government and civil society in this space. No constraints on document dates of publication were made. Included documents were limited to publicly available journal articles, book chapters and institutional reports. In total, 277 documents were extracted, of which 78 were included for review on the basis of the study criteria (see Appendix A).

Five semistructured key informant interviews were conducted via Zoom in January 2023. Using purposive and snowball sampling strategies, key informants were selected on the basis of their involvement and expertise in the area of access to high-cost novel therapies [7]. Consistent with the Research Ethics Board (REB)-approved protocol, an additional five semistructured interviews were conducted in March and April 2025. Key informants included medical activists, pharmaceutical industry representatives, patient advocates, employees at nongovernmental organizations (NGOs), consultants for international organizations and academic researchers. Prior to each interview, key informants provided their written consent to participate. Interviews were conducted in English and were approximately 30–45 min in duration. Interviews were not audio recorded in 2023; however, the interviewer took detailed notes to capture key points throughout each interview. In 2025, interviews were conducted and recorded through Zoom. After each interview, notes were deidentified by the interviewer and transcribed.

Interview notes were coded using a combination of inductive and deductive thematic analysis to identify common themes across the dataset [8]. The initial coding framework was informed deductively by the research project objectives (the role of CSR, government and civil society partnerships in improving access to high-cost novel therapies) and preliminary results from the targeted literature review [8, 9]. The coding framework was refined using inductive thematic analysis, focussing on emerging insights from key informants [9]. Finally, theme memos were developed, with similar codes organized and grouped into potential themes and subthemes by three researchers.

## Results

### Key stakeholders involved in access to high-cost novel therapies

**Fig. 1:**
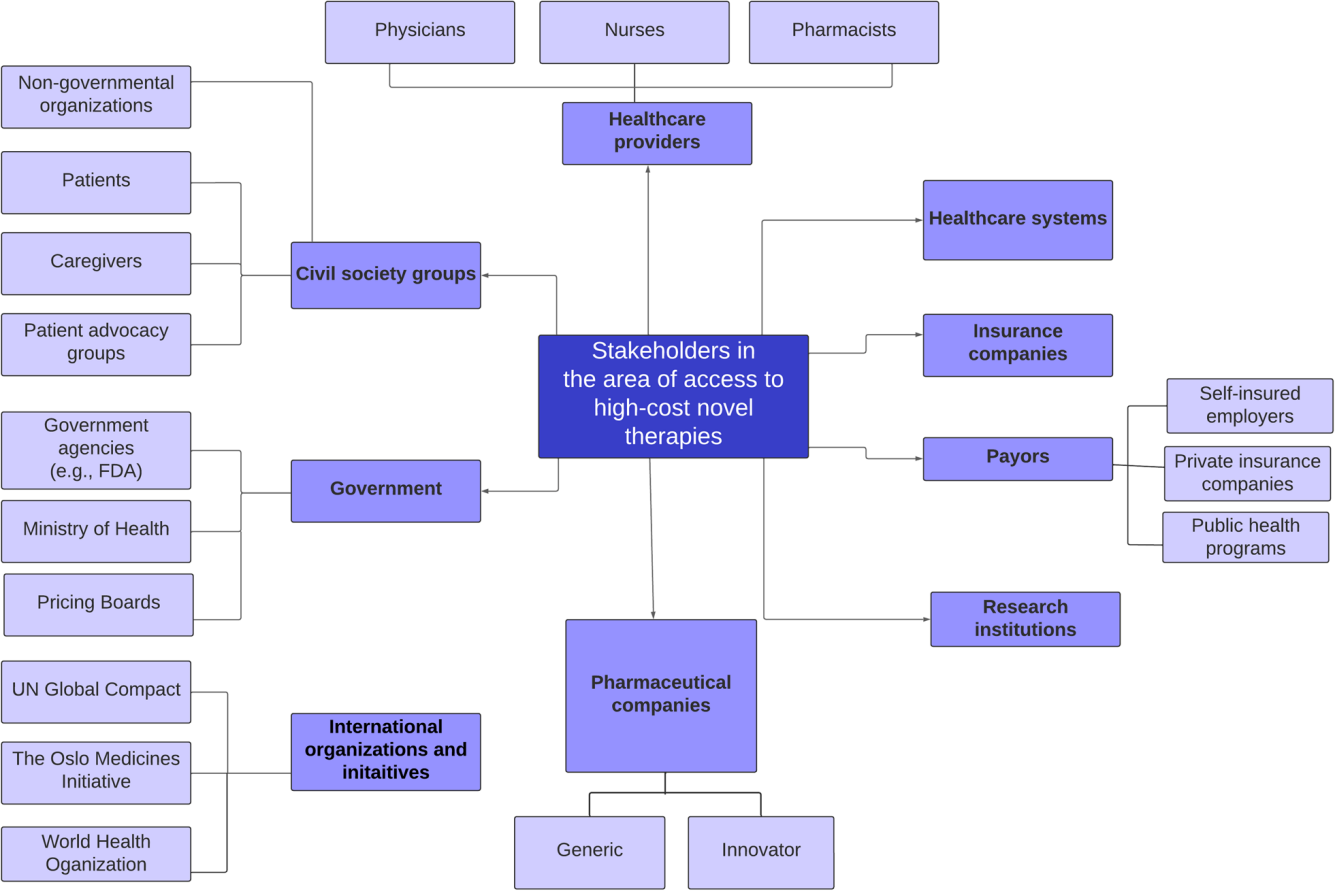
Concept map of key stakeholders involved in access to high-cost medicines

Several key stakeholder groups play a role in mediating patient access to high-cost novel therapies. These are: (i) patients, (ii) civil society organizations (e.g. patient advocacy groups, NGOs), (ii) healthcare providers (e.g. physicians, nurses, pharmacists), (iii) government agencies (e.g. ministries of health, regulatory authorities, pricing boards), (iv) pharmaceutical companies (innovator and generic), (v) research institutions (public and private) and (vii) international organizations (e.g. WHO) [10, 11]. These stakeholders interact with and impact each other to varying degrees, including through government regulation of industry groups, industry manufacturing and supply of medicines, public and private insurance reimbursement of medicines costs incurred by patients and patient advocacy efforts directed towards reducing the cost and increasing the availability of needed medicines (see Fig. 1).

Key informants highlighted significant imbalances in stakeholder power, with pharmaceutical companies having disproportionate access to drug approval and pricing decision-making processes, in stark contrast to the limited leverage held by civil society actors. For example, a key informant involved in patient advocacy for fair access to high-quality and affordable novel therapies stated:

…the extent of the pharmaceutical industry’s influence on government policy in the UK is absolute, from health and regulatory processes to research and health technology assessments. The pharmaceutical industry plays a highly influential role in government agencies’ decisions on what drugs to approve because they provide the agencies with the quality and efficacy of data.

Another key informant similarly commented that pharmaceutical companies are “the most important player in terms of making definitive decisions regarding access to medicines”. These findings reflect a widespread view that pharmaceutical companies have the most influence over access to high-cost novel therapies because they are the stakeholder group most involved in setting research agendas, funding preclinical development and clinical trials and manufacturing new drugs [12, 13]. The informants and literature concluded that pharmaceutical companies wield significant negotiating power over insurance companies and payors when setting the price of novel therapies, which can in turn affect insurance coverage decisions and downstream patient access, particularly amongst those who do not have the economic means to purchase those products out-of-pocket.

Whilst patient advocacy groups and other civil society organizations can help to amplify patient voices, one key informant noted: “Having a patient voice doesn’t necessarily mean it is an independent voice. It is important to be clear about connections between patients and other key stakeholders and the potential conflict of interests”. This comment aligned with statements made by other key informants, who highlighted that whilst some patient advocacy groups operate independently, many adopt advocacy positions aligned with or influenced by their pharmaceutical industry funders [14]. However, they also noted that patient advocacy can have significant beneficial impact for improving patient access to medicines and that there were limited alternative funds to support patient organizations. Many cited human immunodeficiency virus infection/acquired immunodeficiency syndrome (HIV/AIDS) as a disease area where increased research funding and expanded public access to testing and prevention measures arose as a direct consequence of concerted patient advocacy efforts targeting both pharmaceutical companies and governments. Rare diseases were similarly identified as an area with significant patient advocacy efforts to enhance access, with activity directed towards both pharmaceutical companies and governments [15].

### CSR and the pharmaceutical industry

#### Incentives for CSR activity in the pharmaceutical sector

Identified motivations for pharmaceutical sector CSR activities have been grouped into eight key business priorities: (i) building a strong corporate reputation, either at the company or industry level; (ii) improving current market performance; (iii) providing early market intelligence about potential new markets; (iv) encouraging high employee recruitment and retention; (v) encouraging favourable investor and financial analyst outlooks; (vi) reducing operating costs; (vii) reducing onerous regulatory oversight; and (viii) building strong community relationships [16, 17]. The evidence reviewed found that pharmaceutical companies were less likely to engage in CSR activities when they (i) prioritized short-term profits over long-term growth, such as during periods of weak financial performance or in volatile economic environments; (ii) operated in markets with too much or too little competition; or (iii) experienced little normative pressure to engage in responsible business behaviour, either from within the industry or external sources [18–20].

All key informants expressed the view that pharmaceutical companies almost always operate on a profit-driven business model because they are legally required to prioritize shareholder profit expectations. One key informant noted that, “companies are trying to survive and make a profit; they are not charities”. Some of the key informants expressed the opinion that when companies do engage in CSR, it is “mostly window dressing” since drug prices have continued to soar and “even rich countries can no longer pay the prices that are being asked”. One key informant, however, expressed the opinion that CSR does in fact matter for pharmaceutical companies, noting that “it’s quite important from a public relations perspective but oftentimes it’s also very important for employee morale as well”. Still, even though the importance of improving the health and quality of life of patients around the world was generally acknowledged as a central corporate mission aligned with the human right to health, the existence of a binding corporate obligation to engage in CSR activities as part of the international human rights legal framework has consistently been rejected by the industry [21–23]. A key informant further noted that if such a human rights obligation were found to exist, CSR efforts directed towards improving access to medicines would no longer be considered voluntary effort but “a legally binding duty of care under human rights business principles”. The role of government as the primary party responsible for realizing the human right to health was a position adopted by both industry stakeholders in the literature and key informants.

#### Types of CSR activities

A review of the literature identified that pharmaceutical CSR activities directed towards promoting access to medicines primarily involve differential or tiered pricing schemes, patient support programs, medicines donations, medicines delivery programs to remote areas, increased research and development (R&D) investment into rare or neglected diseases and the implementation of flexible patent policies and/or technology transfer programs in developing countries. Though uncommon, some pharmaceutical companies have also adopted a benefit corporation structure, which legally enables them to pursue access to medicines as a corporate goal even if doing so conflicts with the goal of shareholder profit maximization [24]. Differential pricing schemes and flexible patent approaches reviewed in the literature tended to be pursued unilaterally by companies (see Table 1). By contrast, companies were observed to partner with local private sector, government, NGOs and academic actors when implementing patient support programs, medicines donations, medicines delivery programs and rare and neglected R&D initiatives (see Table 2).

**Table 1:** Unilateral CSR strategies for improving access to medicines

CSR strategy	Description
Differential pricing (profit markets versus at-cost markets)	Countries where a pharmaceutical product is sold are assigned to the for-profit market or at-cost market based on income level. At-cost market countries are charged a price equal to the manufacturing costs of the product, without an additional profit-generating markup [25].
Differential pricing (tiered markets)	Countries are assigned to a particular pharmaceutical product pricing tier on the basis of their income levels. As countries experience economic development and growth, they may graduate from a lower pricing tier to a higher pricing tier [25].
Flexible patent approaches (no patent in developing country)	Pharmaceutical companies do not submit a patent application to protect pharmaceutical inventions within developing or least-developed countries. These countries can then manufacture or import generic versions of the product without paying patent royalties or facing patent infringement litigation [25, 26].
Flexible patent approaches (technology transfer)	Pharmaceutical companies actively share key product development know-how with developing country manufacturers to enable local manufacture of their products under a license [26].
Benefit corporation (B-corp certification)	Pharmaceutical company obtains benefit corporation status, which grants it the legal ability to prioritize an access to medicines mission over shareholder profit maximization [27].
Benefit corporation (access to medicines organization as major corporate shareholder)	Pharmaceutical company ensures that one of its major shareholders is an organization with an access to medicines mission, so that shareholder decision-making is directed towards improving patient access to medicines [28].

**Table 2:** CSR strategies pursued in partnership with nonpharmaceutical company actors

CSR partner category	Description
Local private sector (telecommunications company)	Pharmaceutical company partners with private telecommunications company serving local area to deliver mobile health services [17].
Local private sector (banks)	Pharmaceutical company partners with local bank to provide patients with micro-loans as part of a patient support program to finance high-cost medicines purchases [17].
NGOs (health services and pharmaceutical product delivery)	Pharmaceutical company partners with NGO to provide general health services delivery or to ensure delivery of donated medicines to underserved populations [29–31].
NGOs (program financing)	Pharmaceutical company partners with NGO to fund drug delivery, donation, patient financing support or rare/neglected disease R&D programs [16, 31–35].
Intergovernmental organizations (pharmaceutical product donations and delivery)	Pharmaceutical company partners with intergovernmental organization to fund general drug delivery programs for donated products [32, 36].
International organizations (coordinated product price reductions)	Pharmaceutical company partners with international organization to coordinate the implementation of differential or tiered pricing strategies [21].
International organizations (R&D)	Pharmaceutical company partners with international organization to coordinate or fund R&D efforts towards developing medicines for rare or neglected diseases or to promote the development of generic products in developing country markets [29, 32, 34].
Governments (health services capacity and product delivery)	Pharmaceutical company partners with government to build health system capacity or facilitate delivery of donated drugs to a third-party recipient country [27, 32, 35].
Academic institutions (R&D)	Pharmaceutical company partners with an academic research institution to fund or cooperate in R&D for medicines to treat rare or neglected diseases [2, 31, 37].

#### Economy-specific considerations

Pharmaceutical CSR practices directed towards improving access to high-cost medicines varied on the basis of the country and health financing structure in which activities were implemented. Patient support programs, which are designed to reduce the financial barriers to access faced by individual patients on the basis of their personal income level, were found to be more prevalent in high-income countries with minimal state-subsidized healthcare. For example, patient support programs in the United States commonly employ graduated pricing schemes or flat fee reductions to promote access to certain high-cost novel medicines amongst lower-income patients [2, 37]. By contrast, differential and tiered pricing strategies are pursued at the country level to provide entire populations in lower-income countries with access to lower pharmaceutical prices. Though these pricing strategies may improve short-term patient access to medicines in lower-income countries, they have also been criticized for unduly shifting the cost-burden of medicines to middle-income countries, particularly since middle-income countries often do not qualify for additional health financing support from international donors [38, 39]. A key informant engaged in promoting access to low-cost essential medicines in low- and middle-income countries (LMICs) similarly commented:

Products will be made available at cost price and will have no profit for the 45 lowest income countries. But what about middle-income countries? In middle-income countries, a very small proportion of the population is wealthy, and the rest are low income and cannot afford medicines.

Pharmaceutical companies were found to display a greater willingness to engage in CSR strategies involving patent flexibility in lower-income countries compared with higher-income countries [25, 26]. Since patents enable pharmaceutical companies to set higher monopoly prices throughout the life of the patent, this behaviour generally complements differential pricing CSR strategies that focus on setting profit-generating prices in high-income countries rather than low-income countries. Pharmaceutical CSR activity is also more frequently observed in countries with reduced government market intervention activity compared with countries in which government ownership or heavy involvement in the pharmaceutical sector is more prevalent, and in countries with strong cultures of corporate citizenship [38, 40–42]. One key informant noted that “prices are higher if there is a centralized market” and that “a free market encourages competition and lets people set a price that is affordable”. However, for an effective free market, companies and governments need to ensure that competition laws are not violated. For example, another key informant expressed concern that:

Companies protect raw materials and violate competition law. They protect data too. They use these strategies to make products available for extremely high prices. In one country, a company used these strategies to make the price of a drug 500 times higher.

#### Transparency and accountability: setting the CSR agenda and evaluating CSR impact

When setting their CSR agendas, pharmaceutical companies clearly consider the interests and priorities of both their internal and external stakeholders [43]. Internal stakeholders, such as company shareholders and employees, are typically motivated by financial indicators (e.g. profit maximization, greater company capture of market share) or personal alignment with the company’s corporate mission (e.g. developing drugs for patients with rare diseases) [3, 44]. These interests tend to strongly affect the scope of CSR goals pursued by companies, limiting them to activities or disease areas which complement the company’s core business [45]. External stakeholders include direct patient consumers, government payors, civil society groups, NGOs, industry trade associations and media organizations [46]. These stakeholders may voice their concerns either individually or collectively, and can affect a company’s business through direct market activity, regulatory activity or public reputational activity [46]. Government administrative authorities, such as the United States Food and Drug Administration (FDA), European Medicines Agency (EMA) or the United Kingdom Medicines and Healthcare Products Regulatory Agency (MHRA), may also influence the regulatory environment in which pharmaceutical company CSR activities are planned and implemented.

The efficacy of a company’s CSR activities is commonly measured using the evaluative dimensions of centrality, specificity, proactivity, voluntarism and visibility [47]. Centrality describes the measure of closeness of fit between a CSR program and the firm’s mission and objectives. Specificity describes a firm’s ability to capture or internalize the benefits of a CSR program, rather than merely creating collective goods which can be shared by others in the industry, community or society at large. Proactivity describes the degree to which a program is planned in anticipation of emerging social trends and the absence of crisis. Voluntarism describes the scope for discretionary decision-making and the lack of externally imposed compliance requirements. Visibility describes the public recognition a company received by a firm for their CSR activity. All five evaluative metrics can be best met when pharmaceutical companies engage closely with both their internal and external stakeholders to identify common access to medicines priorities that can be effectively met through CSR efforts [48].

Whilst some pharmaceutical industry associations, such as the International Federation of Pharmaceutical Manufacturers and Associations (IFPMA) and European Federation of Pharmaceutical Industries and Associations (EFPIA), have begun developing best practice guidelines for company CSR activities, there are no universally adopted standards for developing CSR programs, engaging in ongoing CSR reporting or evaluating CSR activity in the pharmaceutical sector [34, 49]. Common critiques of pharmaceutical sector CSR agenda development or program evaluation based on company compliance with international corporate reporting standards, such as the UN Global Compact, the Global Reporting Initiative Guidelines, the UN Ruggie Principles or the EU Corporate Governance Code, focus on the generality of these guidelines; since international guidelines are not sector specific, they are unable to provide granular best practices or evaluative metrics for determining a company’s success at enhancing access to high-cost novel medicines through CSR activity [5, 50, 51]. Whilst one key informant noted that international initiatives such as the UN Global Compact can encourage pharmaceutical companies to consider factors such as medicines affordability, availability and access in their operations, another expressed that their compliance record has been inconsistent and that “the UN Ruggie Principles are meant to help guide pharmaceutical companies [to respect human rights], but these companies are not scoring high on this”.

As a result of this lack of standardization in CSR evaluation, civil society benchmarking of pharmaceutical company CSR activities – which involves evaluating companies on the basis of a set of defined CSR metrics then publicly ranking them in order of CSR performance – tend to employ heterogeneous methodologies that are largely dependent on the particular focus of the benchmarking organization. Metrics adopted by the Access to Medicines Index, a well-known and independent CSR benchmarking index of the world’s 20 largest research-based pharmaceutical companies, include company involvement in single-drug donation programs, nonexclusive voluntary licensing agreements, drug donation delivery success, research activity in neglected disease areas and pursuit of not-for-profit pricing strategies [33]. Other metrics that have been used by NGOs (e.g. Oxfam) and academics include company commitment to price transparency, lack of lobbying for stronger intellectual property protections, involvement in joint public–private initiatives, voluntary disclosure of adverse drug reactions and compliance with the UN Sustainable Development Goals [30, 32, 52]. Pharmaceutical sector CSR benchmarking is thought to encourage transparency and accountability within the industry [53]. Proponents also emphasize its ability to promote healthy competition between companies to do better for the public good by not only pressuring them to report their ongoing CSR practices, but also competing against peer companies for favourable public ranking [53]. Industry-specific CSR benchmarking that focusses on improving access to medicines may encourage companies to specifically disclose the CSR activity that is directly relevant to this goal, improving overall disclosure in an area that has historically lacked standardization and providing a mechanism for independent third-party review of companies’ self-published reports [22]. Despite these civil society efforts, however, a lack of standardized CSR reporting and evaluation can nonetheless limit both pharmaceutical companies and other stakeholders’ ability to evaluate the effects of industry-wide CSR efforts on access to high-cost novel therapies, which may in turn undermine the efficient allocation of resources to the CSR activities most likely to improve access for the largest number of patients.

### Limitations to improving access to medicines through CSR initiatives

All key informants expressed the opinion that, in its current form, CSR is not a sustainable or equitable solution in isolation for promoting access to high-cost novel therapies because it reinforces status quo business practices, and thus fails to address systemic causes of access issues. These causes include regulatory and business environments that incentivize companies to pursue pricing strategies that prioritize profit over broad access, low purchasing power amongst public payors in low- and middle-income countries and individual patients who are under- or uninsured, weak supply chains to support delivery to rural or remote areas and reduced R&D for diseases that disproportionately affect patients in low- and middle-income settings.

One key informant expressed:

I don’t believe CSR is going to be relevant or a consequential contributor to access to medicines or affordable medicines… CSR is a weak and fundamentally flawed approach because it will not challenge high drug prices or its root causes.

Another key informant emphasized that CSR activities are not “long-term sustainable solutions”. In reference to one program, which donates millions of tablets a year to prevent a common neglected tropical disease in low-income countries, they remarked:

The donation program required the World Bank to pour a lot of money to continue it. It’s unlikely that we could do the same thing for therapies for breast cancer, asthma or other chronic conditions because there are just too many people affected.

Several key informants noted that without oversight, enforceable baseline standards or clear evaluative metrics, CSR initiatives are primarily used as a way for pharmaceutical companies to continue to advance a profit-driven agenda whilst portraying themselves as responsible actors. One key informant expressed that “CSR is associated with green washing and rights washing, where companies get to pick and choose a cause that is easy for them and will benefit them”. Pharmaceutical companies engaged in CSR were also described as being “unwilling” to disclose the real costs of their products, with key informants noting that even when pharmaceutical companies state that they are devoted to CSR and the public wellbeing, they do not engage in “disclosure and transparency of real costs, making it difficult for the public to obtain fair prices”.

## Discussion

Whilst pharmaceutical companies frequently engage government, NGO and civil society partners when implementing CSR activities, there is a lack of research outlining best practices and opportunities for authentic collaboration between these key stakeholders. Areas of opportunity for further investigation are highlighted below:

### Governments

- Identify how to align reference pricing practices with differential and tiered pricing strategies. Countries with government-subsidized healthcare systems frequently engage in reference pricing when determining the maximum price they are willing to pay a pharmaceutical company for a given product. Reference pricing enables countries to avoid paying substantially more for pharmaceutical products than other reference countries. However, inclusion of a lower-income country within a high- or upper-middle-income country’s list of reference countries may separately disincentivize pharmaceutical companies from including that lower-income country in a lower price tier, since the price given to the reference country affects the price negotiated by the referencing country – even if the referencing country has a higher ability to pay [4, 35].
- Determine how to incentivize the creation of pharmaceutical benefit corporations. The use of benefit corporations in the pharmaceutical sector remains underutilized and understudied, with company benefit corporation status most commonly employed by companies whose primary business involve the sale of consumer goods. In the pharmaceutical sector, where patient brand sensitivity is typically associated with particular medicines rather than manufacturing companies, the absence of consumer demand for benefit corporation manufacturers may contribute to the current lack of pharmaceutical benefit corporations [54]. Governments could consider incentivizing the creation of more pharmaceutical benefit corporations by giving preference to these organizations when distributing grant funding for pharmaceutical R&D initiatives – either generally in the development of high-cost medicines or more specifically in the rare or neglected disease space [55].
- Investigate how to create better models for public–private partnerships for the delivery of drug donations. It is an opportune time to explore the role of public–private partnerships for drug donations that truly help population groups, as WHO is beginning to modernize its drug donation guidelines [56]. High-income countries frequently engage in drug donations as part of larger foreign aid programs. Closer cooperation with pharmaceutical companies to facilitate donations, as well as establishing formal mechanisms between donor and recipient countries with which companies can also engage, can reduce transaction costs associated with the drug donation process. For example, during coronavirus disease 2019 (COVID-19), formal institutional arrangements between donor and recipient governments, Gavi the Vaccine Alliance and COVID-19 vaccine manufacturers through the COVAX Facility facilitated the dissemination of more than 2 billion doses of COVID-19 vaccines to country recipients [52].
- Engage in coordinated strategies for purchasing. Governments may improve access to medicines by pursuing coordinated strategies, such as pooled procurement or partnering with health organization specializing in market shaping activities for pharmaceuticals, to improve access and affordability through market-based mechanisms [57, 58]. If coupled with purchasing criteria that considers a manufacturer’s existing CSR activities with respect to the drug being purchased, this may help incentivize broader manufacturer engagement in CSR activities.

### NGOs and civil society organizations

- Identify strategies and tactics to amplify the voices of underrepresented CSR stakeholders. Pharmaceutical product consumers are a key stakeholder class that companies must consider when developing their CSR agendas [46]. As such, it is pivotal that the priorities of CSR activity beneficiaries, including patients who receive access to high-cost medicines through differential pricing, patient support programs or drug donations, are given adequate consideration when CSR agendas are being drafted by companies. Companies, particularly those with CSR activities that include R&D in the rare and neglected disease space, should take intentional efforts to include the voices of patients who may not be current market consumers. To this end, NGOs and civil society organizations can play a pivotal role in concentrating and amplifying the voices of key CSR stakeholders through patient groups and other patient advocacy efforts. This is particularly important since international initiatives focussed on enhancing access to high-cost therapies coordinated by WHO are governed under its Framework of Engagement with Non-State Actors (FENSA), which one key informant specifically identified as a barrier preventing patient stakeholders from meaningfully participating in their individual capacity in relevant access to medicines dialogues due to the preference that patients be affiliated with larger nonprofit or philanthropic organizations.
- Determine how to measure CSR impact to promote better access to medicines. An important motivating factor for company participation in CSR activity is reputational gain, which is frequently accompanied by the spillover effects of enhanced market performance, greater employee recruitment and retention and improved investor outlook. Government actors and civil society groups alike can bolster this incentive by augmenting the reputational gain available to pharmaceutical companies that engage in meaningful and effective CSR efforts to improve access to high-cost novel medicines. Prizes for the achievement of particular CSR activities, such as company engagement with nonprivate sector partners to develop new therapeutics for rare diseases or provision of technological know-how to developing country manufacturers, can incentivize company appetite for greater CSR activity [15].
- Determine what transparency and accountability mechanisms will generate external demand for CSR activity. Whilst CSR activity may promote the long-term financial growth of a company, it almost always involves short-term costs. This growth is also predicated on the assumption that consumers and other market participants will respond favourably to a company’s CSR activity. If companies face low stakeholder pressure to engage in CSR activity, the financial incentive to assume the associated short-term costs of CSR engagement is reduced [18–20]. Consistent civil society advocacy for pharmaceutical company CSR activity directed towards improving access to high-cost novel medicines, as well as demand for clear corporate disclosure of ongoing and planned CSR activities, can ensure that this external pressure to engage in CSR persists, and thus that the financial and reputational benefits associated with CSR remain available to companies and help them to contribute to critical efforts to improve access to medicines. Key informants also expressed the position that involving civil society organizations can help increase the legitimacy and transparency of policymaking and funding processes initiated by governments related to high-cost novel therapies.

In addition to recommendations focused on advancing CSR, key informants identified areas for further research beyond existing CSR frameworks and towards developing new and sustainable solutions to ensure equitable access to high-cost novel therapies. These include exploring innovative business models that prioritize the human right to health over and above profit maximization, adopting direct regulatory and legislative approaches to enhance access and reduce the cost of novel therapies, committing greater investment in government-sponsored pharmaceuticals research and development and expanding alignment with World Health Assembly (WHA) resolution 72.8 (“Improving the transparency of markets for medicines, vaccines, and other health products”) by conducting independent, real-world studies using empirical data to pinpoint the causal factors underlying the high prices of novel therapies. It is recognized that such efforts may be separately limited by traditionally small shareholder appetite for corporate structures that do not facilitate profit maximization, political inertia and fiscal budget constraints, and the historically limited availability of granular R&D cost information, which has often been treated as confidential information by the pharmaceutical industry [59].

## Conclusions

Timely patient access to safe, effective and quality novel high-cost medicines requires governments and civil society actors to explore new approaches alongside ongoing pharmaceutical industry CSR efforts. In particular, there are opportunities for government, NGO and civil society actors to further promote and enhance the market impact of CSR activity so that it better aligns with patient needs and contributes to the systematic promotion of universal access to lifesaving medicines. Efforts to bolster constructive dialogue and collaboration amongst these stakeholders, enabling all players to better leverage joint expertise and resources, should continue to be pursued in tandem with further research which aims to identify discrete policy directions for future government and civil society action.

## Data Availability

The datasets used and/or analysed during the current study are available from the corresponding author on reasonable request.

## Appendix A

### Titles included in literature review

**Table:**

	Citation information
	Antoun, J. CSR to Increase Access to Medicines: Lessons and Opportunities for the Middle East. in *CSR in the Middle East* 49–71 (Palgrave Macmillan, London, 2012)
	Bae, G., Ahn, J. H., Lim, K. M. & Bae, S. J. Corporate social responsibility of pharmaceutical industry in Korea. *Front Pharmacol* **13**, 3039 (2022)
	Bailey, M. et al. Beyond Philanthropy: the pharmaceutical industry, corporate social responsibility and the developing world. (2002)
	Berete, M. Relationship between corporate social responsibility and financial performance in the pharmaceutical industry. (Walden University, 2011)
	BRATISHKO, Y., Posylkina OV & Kubasova, A. Actual aspects of corporate social responsibility in pharmaceutical companies of Ukraine. *Int Conf Probl Trends Econ Manag Mod World* (2013)
	Brewer, K. Corporate social responsibility in the pharmaceutical industry-Why it matters from business, bioethical and social perspectives. (Wake Forest University, 2014)
	Bruyaka, O., Zeitzmann, H. K., Chalamon, I., Wokutch, R. E. & Thakur, P. Strategic corporate social responsibility and orphan drug development: insights from the US and the EU biopharmaceutical industry. *J Bus Ethics* **117**, 45–65 (2013)
	Capaldi, N. Corporate social responsibility and business ethics in the pharmaceutical industry. In *Innovation and the pharmaceutical industry: Critical reflections on the virtues of profit* (eds. Englehardt, T. & Garrett, J.) (M&M Scrivener Press, 2008)
	Cheah, E. T., Chan, W. L. & Chieng, C. L. L. The corporate social responsibility of pharmaceutical product recalls: an empirical examination of U.S. and U.K. markets. *J Bus Ethics* **76**, 427–449 (2007)
	Cohen-Kohler, J. C. & Illingworth, P. Access to medicines and the role of corporate social responsibility: the need to craft a global pharmaceutical system with integrity. In *The Cambridge Textbook of Bioethics* (eds. Singer, P. & Viens, A.) 359–368 (Cambridge University Press, 2008)
	Cook, L., Lavan, H. & Zilic, I. An exploratory analysis of corporate social responsibility reporting in US pharmaceutical companies. *J Commun Manag* **22**, (2018)
	Czubala, K. Is Access To Medicine A Corporate Social Responsibility? (University of Lund, 2008)
	Danescu, T. & Popa, M.A. Public health and corporate social responsibility: exploratory study on pharmaceutical companies in an emerging market. *Global Health* **16**, 1–9 (2020)
	Daniali, S. M. et al. Evaluation of strategies to improve the corporate social responsibility performance in food and pharmaceutical industries: empirical evidence from Iran. *Sustainability* **13**, 12,569 (2021)
	Demir, M. & Min, M. Consistencies and discrepancies in corporate social responsibility reporting in the pharmaceutical industry. *Sustain Accounting Manag Policy J* **10**, 333–364 (2019)
	Doh, J. P. & Guay, T. R. Corporate social responsibility, public policy, and ngo activism in europe and the united states: an institutional-stakeholder perspective. *J Manag Stud* **43**, 47–73 (2006)
	Droppert, H. & Bennett, S. Corporate social responsibility in global health: an exploratory study of multinational pharmaceutical firms. *Global Health* **11**, 1–8 (2015)
	Dunfee, T. W. Do firms with unique competencies for rescuing victims of human catastrophes have special obligations? Corporate responsibility and the AIDS catastrophe in Sub-Saharan Africa. *Bus Ethics Q* **16**, 23–51 (1991)
	Eiser, A. R. & Field, R. I. Can benefit corporations redeem the pharmaceutical industry? *Am J Med* **129**, 651–652 (2016)
	Fischer, A., Dewatripont, M. & Goldman, M. Benefit corporation: a path to affordable gene therapies? *Nat Med 2019 2512* **25**, 1813–1814 (2019)
	Forman, L. & Clare Kohler, J. Chapter 1: Introduction. in *Access to Medicines as a Human Right: Implications for Pharmaceutical Industry Responsibility* (eds. Forman, L. & Kohler, J.) (University of Toronto Press, 2012)
	Fu, L., Boehe, D. & Orlitzky, M. Are R&D-Intensive firms also corporate social responsibility specialists? A multicountry study. *Res Policy* **49**, 104,082 (2020)
	Giovambattista Calafatti, S. Orphan drugs and corporate social responsibility: which possible future scenarios? (Luiss Guido Garli Libera Universita Internazionale Delgi Studi Sociali, 2014)
	Givel, M. Modern neoliberal philanthropy: motivations and impact of Pfizer Pharmaceutical’s corporate social responsibility campaign. 10.1080/01436597.2012.755013**34**, 171–182 (2013)
	Harris, R. *Oslo Medicines Initiative Policy Brief*. https://www.who.int/europe/publications/i/item/WHO-EURO-2022-6008-45773-65874 (2022)
	Hurst, D. J. Restoring a reputation: invoking the UNESCO Universal Declaration on Bioethics and Human Rights to bear on pharmaceutical pricing. *Med Heal Care Philos* **20**, 105–117 (2017)
	Khan, Y., Rehman, D. A., Shah, T. U. & Khan, K. The impact of corporate social responsibility on firm’s productivity: a comparative study of two competing firms having UN Global Compact Status. *The Discourse* **4**, 1–16 (2018)
	Khanna, A. Pharmaceutical industry’s corporate social responsibility towards HIV/AIDS. *J Postgrad Med* **52**, 194 (2006)
	Knudsen, J. S. Company Delistings from the UN Global Compact: limited business demand or domestic governance failure? *J Bus Ethics* **103**, 331–349 (2011)
	Lee, H., Kim, S. Y., Kim, G. & Kang, H. Y. Public preferences for corporate social responsibility activities in the pharmaceutical industry: Empirical evidence from Korea. *PLoS One* **14**, e0221321 (2019)
	Lee, M. & Kohler, J. Benchmarking and transparency: Incentives for the pharmaceutical industry’s corporate social responsibility. *J Bus Ethics* **95**, 641–658 (2010)
	Leisinger, K. M. & Wagner, A. K. Access to Medicines and Corporate Social Responsibilities of the Pharmaceutical Industry. (2013)
	Leisinger, K. M. Opportunities and risks of the United Nations Global Compact: the Novartis Case Study. *J Corp Citizsh* **11**, (2003)
	Leisinger, K. M. The corporate social responsibility of the pharmaceutical industry: idealism without illusion and realism without resignation. *Bus Ethics Q* **15**, 577–594 (2005)
	Malik, M. S. & Kanwal, L. Impact of corporate social responsibility disclosure on financial performance: case study of listed pharmaceutical firms of Pakistan. *J Bus Ethics* **150**, 69–78 (2018)
	Min, M., Desmoulins-Lebeault, F. & Esposito, M. Should pharmaceutical companies engage in corporate social responsibility? *J Manag Dev* **36**, (2017)
	Moon, W. Corporate social vs. developmental responsibility: corporate citizenship in the restructuring of China’s pharmaceutical industry. 10.1080/13621025.2020.1812954**24**, 887–903 (2020)
	Nussbaum, A. K. Ethical corporate social responsibility (CSR) and the pharmaceutical industry: a happy couple? 10.1057/jmm.2008.33**9**, 67–76 (2009)
	O’riordan, L. & Fairbrass, J. Corporate social responsibility (CSR): models and theories in stakeholder dialogue. (2008) 10.1007/s10551-008-9662-y
	Oke, E. K. Using the right to health to enforce the corporate responsibilities of pharmaceutical companies with regard to access to medicines. *J Heal Dipl* **1**, (2013)
	Oshionebo, E. The U.N. Global Compact and accountability of transnational corporations: separating myth from realities. *Fla J Int Law* **19**, (2007)
	Osuji, O. K. & Umahi, O. T. Pharmaceutical companies and access to medicines – Social integration and ethical CSR resolution of a global public choice problem. 10.1080/17449626.2012.702678**8**, 139–167 (2012)
	Outterson, K. & Rex, J. H. Evaluating for-profit public benefit corporations as an additional structure for antibiotic development and commercialization. *Transl Res* **220**, 182–190 (2020)
	Petkoski, D. & Twose, N. *Public policy for corporate social responsibility*. (2003)
	Piddock, L. J. V. et al. A nonprofit drug development model is part of the antimicrobial resistance (AMR) solution. *Clin Infect Dis* **74**, 1866–1871 (2022)
	Pitts, P. J. Associate editor’s commentary: measuring responsibility. (2013) 10.1177/2168479013483135
	Rahman, M., Chowdhury, S., Zaleha, A. S. & Rasid, A. Corporate social responsibility and firm financial performance: Bangladeshi listed pharmaceutical companies. *Int J Emerg Technol Innov Res* **7**, (2020)
	Rana, I. & Asad, F. Impact of corporate social responsibility on financial performance evidence from pharmaceutical sector listed companies of Pakistan. *Eur Bus Manag* **4**, 1 (2017)
	Rashmi, R. Corporate social responsibility of pharmaceutical industry towards access to medicine: a case study of GlaxoSmithKline. In *Corporate Social Responsibility: Concepts, Methodologies, Tools, and Applications* (Information Resources Management Association, 2019)
	Reis, E. Corporate Social Responsibility Practices of Pharmaceutical Companies in China: A Scale Development and Empirical Study. (ISCTE Business School, 2016)
	Rimmer, M. The Lazarus effect: the (RED) campaign and creative capitalism. in *Incentives for Global Public Health: Patent Law and Access to Essential Medicines* (eds. Pogge, T., Rimmer, M. & Rubenstein, K.) 313–340 (Cambridge University Press, 2010)
	Ritter, G. S. Are drug companies living up to their human rights responsibilities? The Merck perspective. *PLOS Med* **7**, e1000343 (2010)
	Rocha, M. de M., de Andrade, E. P., Alves, E. R., Cândido, J. C. & Borio, M. de M. Access to innovative medicines by pharma companies: sustainable initiatives for global health or useful advertisement? 10.1080/17441692.2020.1729391**15**, 777–789 (2020)
	Rose, J., Quave, C. L. & Islam, G. The four-sided triangle of ethics in bioprospecting: pharmaceutical business, international politics, socio-environmental responsibility and the importance of local stakeholders. *Ethnobiol Conserv* **1**, (2012)
	Rusu, A., Kuokkanen, K. & Heier, A. Current trends in the pharmaceutical industry – A case study approach. *Eur J Pharm Sci* **44**, 437–440 (2011)
	Salton, R. & Jones, S. The corporate social responsibility reports of global pharmaceutical firms. *Br J Healthc Manag* **21**, (2015)
	Saxena, K., Balani, S., Pradesh, U. & Srivastava, P. The relationship among corporate social responsibility, sustainability and organizational performance in pharmaceutical sector: a literature review. *Int J Pharm Healthc Mark* **15**, 572–597 (2021)
	Silva Filho, C.F., Benedicto, S.C. De, Sugahara, C.R. & Georges, M.R.R. Corporate social responsibility of pharmaceutical industry in Brazil. *Int. J. Humanit. Soc. Sci.* **9**, (2019)
	Slob, B. & Weyzig, F. Corporate lobbying and corporate social responsibility: aligning contradictory agendas. in *Business, Politics and Public Policy* 160–183 (Palgrave Macmillan, London, 2010). 10.1057/9780230277243_7
	Smith, A. D. Corporate social responsibility practices in the pharmaceutical industry. *Bus Strateg Ser Northampt* **9**, (2008)
	Snider, J., Hill, R. P. & Martin, D. Corporate social responsibility in the twenty-first century: a view from the world’s most successful firms. *J Bus Ethics* **48**, 175–187 (2003)
	Sung, M. et al. Pharmaceutical industry’s engagement in the global equitable distribution of covid-19 vaccines: corporate social responsibility of EUL vaccine developers. *Vaccines* **9**, 1183 (2021)
	Tavis, L. A. Novartis and the U.N. Global Compact Initiative. *Vanderbilt J. Transnatl. Law* **36**, (2003)
	Theron, D. Corporate social responsibility: a pharmaceutical industry analysis. *Acta Commer* **5**, (2005)
	Thorsteinsdóttir, H., Ovtcharenko, N. & Kohler, J. C. Corporate social responsibility to improve access to medicines: the case of Brazil. *Global Health* **13**, 1–11 (2017)
	Ulfa, R., Amalia, N. I., Rajagukguk, M. & Muda, I. Creating shared value by integrating sustainable objective to the next level for the Kimia Farma Pharmaceuticals Firm. *J Pharm Negat Results* **13**, (2022)
	van de Pol, P. K. C. & de Bakker, F. G. A. Direct-to-consumer advertising of pharmaceuticals as a matter of corporate social responsibility? *J Bus Ethics* **94**, 211–224 (2010)
	van den Belt, H. Intellectual property and global health: from corporate social responsibility to the access to knowledge movement. *Liverp Law Rev* **34**, 47–73 (2013)
	Verweij, M. Case description: the pharmaceutical industry and the AIDS crisis. *Issues Bus Ethics* **28**, 33–45 (2011)
	Vian, T. Corporate Social responsibility in global health: the Pfizer Global Health Fellows International Volunteering Program. *Hum Resour Plan* **30**, (2007)
	Vir, A., Kanwar, S. & Rahim, M. M. Social Responsibilities of the global pharmaceuticals companies Kanwar and Rahim social responsibilities of the global pharmaceuticals companies: towards an ethical health care paradigm. *J Law Med* **26**, 750–763 (2019)
	Vitezić, N. A Measurement system of corporate social responsibility in the pharmaceutical industry of the region. *Int J Manag Inf Syst Quart* **14**, 57 (2010)
	Volodina, A., Sax, S. & Anderson, S. Corporate Social responsibility in countries with mature and emerging pharmaceutical. *Pharm Pract (Granada)* **7**, 228–237 (2009)
	Wan, A. X., Zhong, Z., Zheng, C., Zhao, X. & Elahi, E. Use of cloud model to evaluate corporate social responsibility of pharmaceutical companies. *Kybernetes* **51**, 896–915 (2022)
	West, T. Corporate social responsibility within the pharmaceutical industry. (University of Saskatchewan, 2012)
	Witkowska, J. Corporate social responsibility (CSR) of innovative pharmaceutical corporations. The case of BIOGEN. *Comp Econ Res* **21**, 45–62 (2018)
	Yang, M., Wang, J., Maresova, P. & Akbar, M. Can the spending of corporate social responsibility be offset? Evidence from pharmaceutical industry. *Econ Res* **35**, (2022)
	Yıldırım, M. & Dinçer, M. A. M. How the process of the CSR activities works on private hospitals and pharmaceutical firms: multiple case study from strategic perspective. *J Relig Health* **59**, 961–985 (2020)

## Appendix B

### Interview guide

1. Please describe your responsibilities at your organization. Please describe the work that you do and how you are engaged in the area of access to high-cost novel therapies.

2. Are you familiar with the Oslo Medicines Initiative?

If yes: What do you think are the key outcomes of the Oslo Initiative and why?

If no, interviewer describes the OMI to the key informant then asks: What do you think.

are the key outcomes of the Oslo Initiative and why?

3. Based off what you know about the OMI, what would you hope for the OMI to achieve?

4. Who are the key stakeholders in access to high-cost novel therapies?

5. What is the role of governments in ensuring access of its populations to high-cost novel therapies, particularly in the context of corporate social responsibility in this area?

6. What is the role of the private sector in ensuring access of populations to high-cost novel therapies, particularly in the context of corporate social responsibility in this area?

7. What is the role of civil society in helping to ensure access to high-cost novel therapies? What civil society group has been most successful in this area and why?

8. What are the factors that hinder or facilitate access to high-cost novel therapies?

9. What do you think is needed to improve access of the global population to high-cost novel therapies?

10. What research questions do you think need to be addressed for policy impact related to high-cost medicines and access?

11. What other issues do you think are important to explore further in this area?

## Abbreviations

CSR: Corporate social responsibility
EFPIA: European Federation of Pharmaceutical Industries and Associations
EMA: European Medicines Agency
EU: European Union
FDA: Food and Drug Administration
IFPMA: International Federation of Pharmaceutical Manufacturers and Associations
LMIC: Low- and middle-income country
MHRA: Medicines and Healthcare products Regulatory Authority
NGO: Nongovernmental organization
NMP: Novel Medicines Platform
OMI: Oslo Medicines Initiative
R&D: Research and development
UN: United Nations
WHO: World Health Organization
